# FOOD INSECURITY AMONG OLDER ADULTS WITH A HISTORY OF INCARCERATION: A MIXED METHODS STUDY

**DOI:** 10.1093/geroni/igac059.1048

**Published:** 2022-12-20

**Authors:** Rodlescia Sneed, Tamara Jordan

**Affiliations:** Wayne State University, Detroit, Michigan, United States; Michigan State University College of Human Medicine, Flint, Michigan, United States

## Abstract

The purpose of this study was to use a mixed methods approach to describe the association between history of incarceration (HOI) and food insecurity (FI) among older adults. Quantitative data were obtained from the Health and Retirement Study, a population-based study of community-dwelling adults (n=12,702) aged >50. Qualitative data were obtained via key informant interviews with formerly incarcerated older adults and the human service providers serving them (n=15). Multiple logistic regression was used to estimate the association between HOI and FI, adjusting for demographic variables. HOI was associated with increased odds of FI (OR 1.83; 95% CI 1.52-2.21). Race/ethnicity moderated the association between history of incarceration and food insecurity, with effects observed among Non-Hispanic Blacks (OR 1.78; 95% CI 1.29-2.46) and Whites (OR 2.27; 95% CI 1.74-2.97), but not Hispanics (OR 1.11; 95% CI 0.69-1.77) or those of other racial/ethnic groups (OR 1.79; 95% CI 0.71-4.52). Explanations for the association between HOI and FI obtained from qualitative interviews included ineligibility for food assistance programs due to felony conviction, lack of safe places to store healthy food, and difficulty using technologies needed to enroll in food assistance programs. The most common barrier associated with inaccessibility to healthful foods for this population, according to interviewees, is ineligibility for food assistance programs. FI is an important issue among older adults with a HOI. Re-examination of policies and procedures for accessing food assistance programs may be needed to reduce FI in this population.

